# Gut Microbiota in the First 2 Years of Life and the Association with Body Mass Index at Age 12 in a Norwegian Birth Cohort

**DOI:** 10.1128/mBio.01751-18

**Published:** 2018-10-23

**Authors:** Maggie A. Stanislawski, Dana Dabelea, Brandie D. Wagner, Nina Iszatt, Cecilie Dahl, Marci K. Sontag, Rob Knight, Catherine A. Lozupone, Merete Eggesbø

**Affiliations:** a Department of Epidemiology, Colorado School of Public Health, Aurora, Colorado, USA; b Department of Pediatrics, University of Colorado School of Medicine, Aurora, Colorado, USA; c Department of Biostatistics and Informatics, Colorado School of Public Health, Aurora, Colorado, USA; d Department of Environmental Exposure and Epidemiology, Norwegian Institute of Public Health, Oslo, Norway; e Department of Community Medicine and Global Health, University of Oslo, Oslo, Norway; f Center for Public Health Innovation, CI International; g Department of Pediatrics, University of California San Diego, La Jolla, California, USA; h Department of Computer Science and Engineering, University of California San Diego, La Jolla, California, USA; i Center for Microbiome Innovation, University of California San Diego, La Jolla, California, USA; j Department of Biomedical Informatics and Personalized Medicine, University of Colorado School of Medicine, Aurora, Colorado, USA; Yale School of Public Health

**Keywords:** children, infants, microbiota, obesity

## Abstract

Childhood obesity is a growing problem worldwide. Recent research suggests that the gut microbiota may play an important and potentially causal role in the development of obesity and may be one mechanism that explains the transgenerational transmission of obesity risk. Here we examine the early-life gut microbiota at days 4, 10, 30, 120, 365, and 730 and the association with body mass index (BMI) z-scores at age 12 in a Norwegian prospective cohort (*n* = 165), and evaluate how these BMI-associated taxa relate to maternal overweight/obesity (Ow/Ob) and excessive gestational weight gain (GWG). We performed 16S rRNA gene sequencing on the gut microbiota samples. Taxonomic phylogeny at days 10 and 730 was significantly associated with childhood BMI, and the gut microbiota taxa at two years of age explained over 50% of the variation in childhood BMI in this cohort. The subset of the early-life taxa within the gut microbiota that best predicted later childhood BMI showed substantial overlap with the maternal taxa most strongly associated with maternal Ow/Ob and excessive GWG. Our results show an association between the infant gut microbiota and later BMI, and they offer preliminary evidence that the infant gut microbiota, particularly at 2 years of age, may have potential to help identify children at risk for obesity.

## Abstract (precis)

Understanding the role of the early-life gut microbiota in obesity is important because there may be opportunities for preventive strategies. We examined the relationships between infant gut microbiota at six times during the first two years of life and BMI at age 12 in a birth cohort of 165 children and their mothers. We found that the gut microbiota from early life to two years shows an increasingly strong association with childhood BMI. This study provides preliminary evidence that the gut microbiome at 2 years of age may offer useful information to help to identify youth who are at risk for obesity, which could facilitate more-targeted early prevention efforts.

## INTRODUCTION

The prevalence of childhood obesity has been increasing in most nations across the globe in recent decades and is considered an epidemic (1, 2). Although we know that human genetics and aspects of the “Western lifestyle” including poor diet and lack of exercise contribute to this epidemic, our understanding of the physiological etiology of obesity and effective ways to curb obesity in children is incomplete (1).

In recent decades, the idea that the gut microbiota may play an important and potentially causal role in obesity has gained traction. Differences in gut microbiota have been associated with overweight/obesity in both adults and children, and there is growing evidence of possible causal mechanisms (3–5). The role of early-life gut microbiota in the development of obesity is of particular interest because of the potential application to prevention efforts. Taxonomic differences have been noted at different time points during the first 2 years of life with measures of weight or adiposity at various times during the first years of life through early childhood (6–9). Our prior work in this cohort also showed that early gut microbiota was associated with infant growth velocity, a risk factor for later obesity (10). Exposures that alter the early infant gut microbiota, such as antibiotic use (11, 12) and delivery via cesarean section (13), have also been associated with childhood obesity risk. Furthermore, research in animal models has provided compelling evidence that perturbations in the early-life gut microbiota has long-term metabolic consequences (14).

The early-life gut microbiota is highly dynamic and shaped by many factors, such as maternal gut microbiota, delivery mode, breastfeeding, and antibiotics, making this a particularly challenging time period to study (15–17). Many of the studies in this area have had small sample sizes, only one or two time periods of gut microbiota data collection, or only early measures of child weight, which correlates with later obesity but not as strongly as measures later in childhood (18). Some of these studies have also focused exclusively on subsets of children with similar early-life exposures (e.g., full term, vaginally born, unexposed to antibiotics, etc.), but detailed information about early-life exposures is not always known by clinicians. Thus, it is also important to gain a better understanding of the longitudinal association between gut microbiota and obesity and whether the association is evident across diverse early-life exposures. In this study, we examine the gut microbiota at 6 time points over the first 2 years of life and the relationship with age- and sex-specific body mass index (BMI) z-scores at age 12 in a Norwegian birth cohort (NoMIC), both overall and when accounting for diverse early-life exposures, including differences in gestational age, antibiotic use, delivery mode at birth, twin status, and breastfeeding. We also examine the roles of maternal overweight/obesity (Ow/Ob), excessive gestational weight gain (GWG), and maternal gut microbiota in this association. This is an important area of research because an early marker of obesity risk could allow for more-targeted prevention efforts in children or pregnant women (19).

## RESULTS

### Characterization of the infants and their gut microbiota during the first 2 years of life.

Our primary analysis includes gut microbiota data from 165 infants. At 12 years of age, 20% (*n* = 33) of the children were overweight or obese; the median (IQR) age- and sex-specific BMI z-score at this age was 0.1 (−0.5 to 0.7) (Table 1). Mothers of Ow/Ob children tended to have less education and higher prepregnancy BMI than mothers of non-Ow/Ob children; they also had higher rates of smoking during pregnancy and shorter duration of breastfeeding (Table 1).

**TABLE 1:** Characteristics and early-life exposure of infants in the NoMIC cohort^*a*^

Characteristic	Median (IQR) or *n* (%)			*P* value
	Total (*n* = 165)	Non-Ow/Ob = 0 (*n* = 132)	Ow/Ob (*n* = 33)	
Parental characteristics				
Maternal age (yr)	30 (27–32)	30 (28–32)	29 (26–33)	0.45
Ethnic Norwegian	144 (87.3%)	116 (87.9%)	28 (84.8%)	0.26
Missing	5 (3.0%)	3 (2.3%)	2 (6.1%)	
Maternal education				
<12 yr education	15 (9.1%)	10 (7.6%)	5 (15.2%)	0.002
12 yr education	29 (17.6%)	20 (15.2%)	9 (27.3%)	
>12 yr education	118 (71.5%)	99 (75.0%)	19 (57.6%)	
Missing	3 (1.8%)	3 (2.3%)	0 (0.0%)	
Maternal prepregnancy BMI	24.5 (21.4–27.1)	23.1 (21.0–26.1)	26.3 (24.2–30.1)	<0.001
Maternal prepregnancy Ow/Ob	73 (44.2%)	49 (37.1%)	24 (72.7%)	<0.001
Paternal BMI	26.1 (23.7–28.2)	25.4 (23.7–27.7)	27.8 (25.9–29.2)	0.13
Exposures during pregnancy				
Maternal smoking during pregnancy	20 (12.1%)	12 (9.1%)	8 (24.2%)	0.02
Diabetes				
Type 1	1 (0.6%)	1 (0.8%)	0 (0.0%)	0.64
Gestational diabetes	1 (0.6%)	1 (0.8%)	0 (0.0%)	
High BP	9 (5.5%)	7 (5.3%)	2 (6.1%)	1.00
Parity				
No prior pregnancies	75 (45.5%)	61 (46.2%)	14 (42.4%)	0.17
1 prior child	54 (32.7%)	46 (34.8%)	8 (24.2%)	
>1 prior child	36 (21.8%)	25 (18.9%)	11 (33.3%)	
Infant and birth				
Female sex	75 (45.5%)	59 (44.7%)	16 (48.5%)	0.70
Twins	21 (12.7%)	20 (15.2%)	1 (3.0%)	0.08
Gestational age at birth (wk)	39.0 (36.0–40.0)	39.0 (36.5–40.0)	39.0 (36.0–40.0)	0.78
C-section delivery	51 (30.9%)	38 (28.8%)	13 (39.4%)	0.24
Birth weight (g)	3,290 (2,560–3,750)	3,260 (2,540–3,740)	3,370 (2,878–3,990)	0.31
Infant feeding				
Length of any breastfeeding (mo)	10 (5–13)	11 (5.5–14)	7 (3–13)	0.03
Length of exclusive breastfeeding (mo)	4 (2–6)	5 (2–6)	2 (0–5)	0.01
Child age when introduced to porridge (wk)	19 (16–22.5)	20 (16–23)	18 (16–20)	0.24
Child age when introduced to solids (wk)	20 (16–26)	22 (17.5–26)	18 (16–26)	0.12
Antibiotic exposures				
Maternal antibiotics during pregnancy	56 (33.9%)	46 (34.8%)	10 (30.3%)	0.83
Missing	5 (3.0%)	3 (2.3%)	2 (6.1%)	
Antibiotics given to newborn	24 (14.5%)	16 (12.1%)	8 (24.2%)	0.09
Missing	2 (1.2%)	1 (0.8%)	1 (3.0%)	
Child antibiotics before	10 (6.1%)	6 (4.5%)	4 (12.1%)	0.11
4 days				
10 days	13 (7.9%)	9 (6.8%)	4 (12.1%)	0.30
30 days	18 (10.9%)	13 (9.8%)	5 (15.2%)	0.38
120 days	25 (15.2%)	20 (15.2%)	5 (15.2%)	1.00
1 year	68 (41.2%)	52 (39.4%)	16 (48.5%)	0.34
2 years	93 (56.4%)	74 (56.1%)	19 (57.6%)	0.88
Childhood BMI				
BMI-for-age Z	0.1 (–0.5 to 0.7)	–0.1 (–0.6 to 0.4)	1.7 (1.4–1.9)	<0.001
BMI-for-age percentile	54.3 (30.2–77.2)	47.6 (26.9–64.4)	95.1 (92.0–97.1)	<0.001

a Children are grouped by overweight/obesity (Ow/Ob) status at age 12, as defined by age- and sex-specific BMI percentiles of ≥85th percentile.

Figure S1 in the supplemental material shows the gut microbiota taxonomic composition of the infants at six time points during the first 2 years of life (days 4, 10, 30, 120, 365, and 730), as well as that of their mothers at the time of delivery, by the child’s Ow/Ob status at age 12. The composition changed significantly over time, at both the phylum and genus levels. The infant samples progressed toward a more adult-like community with age, as seen by comparing the infants to the maternal samples in the taxonomy plots (Fig. S1), as well as in the principal coordinate analysis plots of UniFrac distance (Fig. S2). This progression of infant gut microbiota composition toward an adult-like community is typical and usually occurs around the age of 2 to 3 years (20, 21).

10.1128/mBio.01751-18.1 FIG S1 Characterization of the gut microbiota samples of mothers at the time of delivery and infants over the first two years of life. Mean relative abundance of the most prevalent phyla (top) and genera (bottom) in maternal gut microbiota samples at the time of delivery (*n* = 71; labeled “Mother”) and infant gut microbiota samples over the first two years of life by overweight/obese (Ow/Ob) status at age 12 and sampling time (4, 10, 30, 120, 365, and 730 days with *n *= 147, 151, 155, 147, 118, and 63, respectively). Download FIG S1, EPS file, 0.04 MB. Copyright © 2018 Stanislawski et al. 2018 Stanislawski et al. This content is distributed under the terms of the Creative Commons Attribution 4.0 International license.

10.1128/mBio.01751-18.2 FIG S2 Principal coordinate analysis plots of weighted (left) and unweighted (right) UniFrac distance of infant and maternal samples by sampling time. The number of samples varied with sampling time: 4 days, *n* = 147; 10 days, *n* = 151; 30 days, *n* = 155; 120 days, *n* = 147; 365 days, *n* = 118; 730 days, *n* = 63; and maternal samples, *n* = 71. Download FIG S2, JPG file, 0.02 MB. Copyright © 2018 Stanislawski et al. 2018 Stanislawski et al. This content is distributed under the terms of the Creative Commons Attribution 4.0 International license.

### Infant gut microbiota, particularly at 2 years of age, is predictive of childhood BMI z-score.

**FIG 1:**
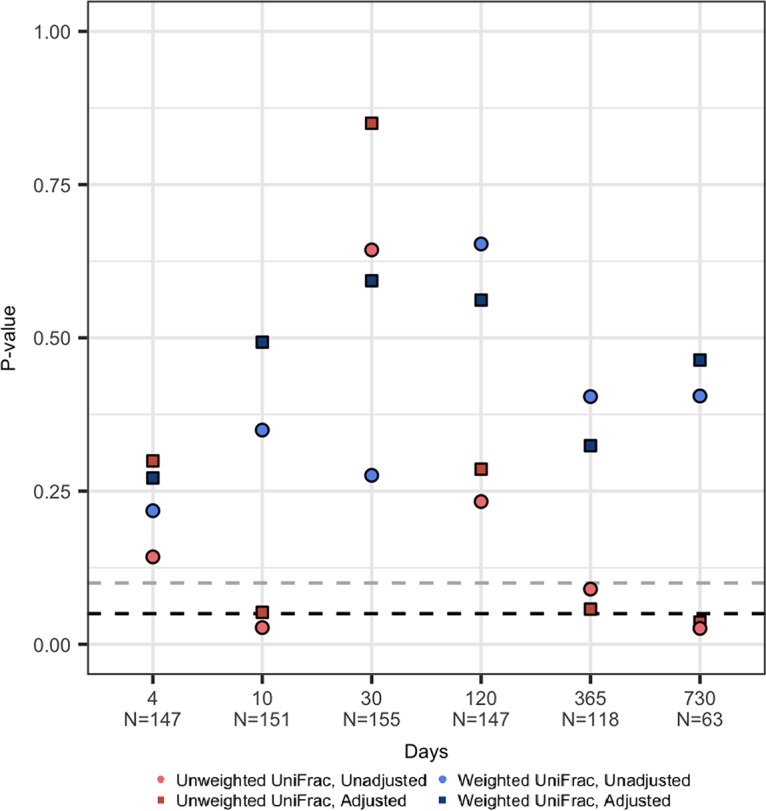
Evaluation of the association between the gut microbiota taxonomic composition at 6 time points in early life with BMI z-score at age 12. This plot shows the estimated *P* values from unadjusted (circles) and adjusted (squares) Microbiome Regression-Based Kernel Association Tests of the unweighted (coral) and weighted (blue) UniFrac distance matrices and BMI z-scores at age 12. These UniFrac measures capture qualitative and quantitative differences in phylogeny, respectively. Dashed lines show *P* = 0.05 (black) and *P* = 0.1 (gray). Adjusted models controlled for breastfeeding, delivery mode, antibiotic exposures, gestational age at birth, and twin status.

**FIG 2:**
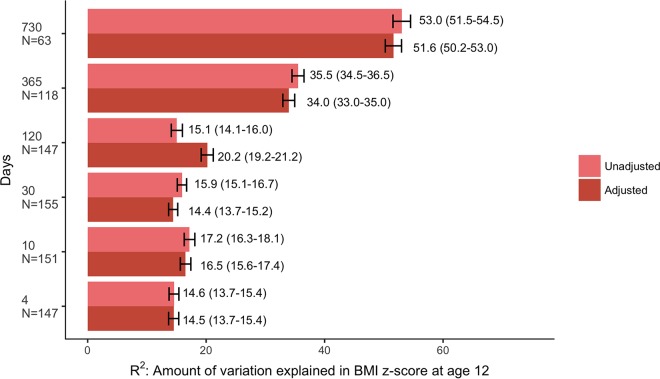
Amount of variation in BMI z-score at age 12 explained by the infant gut microbiota at each sampling time. This plot shows the estimated *R*^2^ values with 95% confidence intervals from the repeated cross-validation of the random forests to predict childhood BMI z-scores based on infant gut microbiota characteristics at each sampling time, both unadjusted and adjusted for confounding factors (breastfeeding, delivery mode, antibiotic exposures, gestational age at birth, and twin status).

The overall infant gut microbiota taxonomic phylogeny (unweighted UniFrac) at days 10 and 730 was significantly associated with sex- and age-specific BMI z-scores at age 12 (Fig. 1). Since it is possible a specific subset of the taxa could be predictive of childhood BMI even though the overall composition is not significantly associated, we evaluated whether gut microbiota taxa at each sampling time during the first 2 years of life predicted later BMI using random forests (22). As can be seen in Fig. 2, the gut microbiota of infants during the first 4 months explained a significant portion of the variation in BMI z-score (*R*^2^ values range from 14.6% to 15.1%). This association strengthened with age, and more than half (53.0%) of the variability in BMI z-scores at 12 years of age was explained by the gut microbiota composition at 2 years of age (Fig. 2). This is substantially more than other predictors of child BMI; for example, taken together child BMI predictors such as delivery mode, exclusive breastfeeding duration, antibiotic exposure, twin status, gestational age at birth, and maternal factors (including prepregnancy BMI, smoking during pregnancy, and education) explained 15.2% of the variation in child BMI z-score. Interestingly, the confounding variables of delivery mode, exclusive breastfeeding duration, antibiotic exposure, twin status, and gestational age included in the random forests were not among the most important predictors of later BMI (see Fig. S3 for a conceptual model and description of our choice of confounding variables); we estimated *R*^2^ values of the random forests both with and without these confounding factors (Fig. 2), and the values were comparable.

10.1128/mBio.01751-18.3 FIG S3 Conceptual model and direct acyclic graphs (DAGs) of the relationships examined in this study. The diagram at the top shows the overall conceptual model of the relationships examined in this study between maternal overweight/obesity (Ow/Ob) and excessive gestational weight gain (GWG), maternal and infant gut microbiota, and childhood BMI. Below this are DAGs of the relationships between exposures, outcomes, and other factors in the analysis of (i) infant gut microbiota and BMI z-score at age 12 and (ii) maternal characteristics and infant gut microbiota. In prior work, we examined the relationship between maternal Ow/Ob and excessive GWG and maternal gut microbiota at the time of delivery. DAGs show the conceptual relationships between exposures, outcomes, confounders, and mediators and inform decisions about what to include in statistical models. Relationships were determined based on prior research when available and on univariate analyses in this cohort for factors without prior research (using a *P* value cutoff of 0.1). Download FIG S3, TIF file, 0.8 MB. Copyright © 2018 Stanislawski et al. 2018 Stanislawski et al. This content is distributed under the terms of the Creative Commons Attribution 4.0 International license.

**FIG 3:**
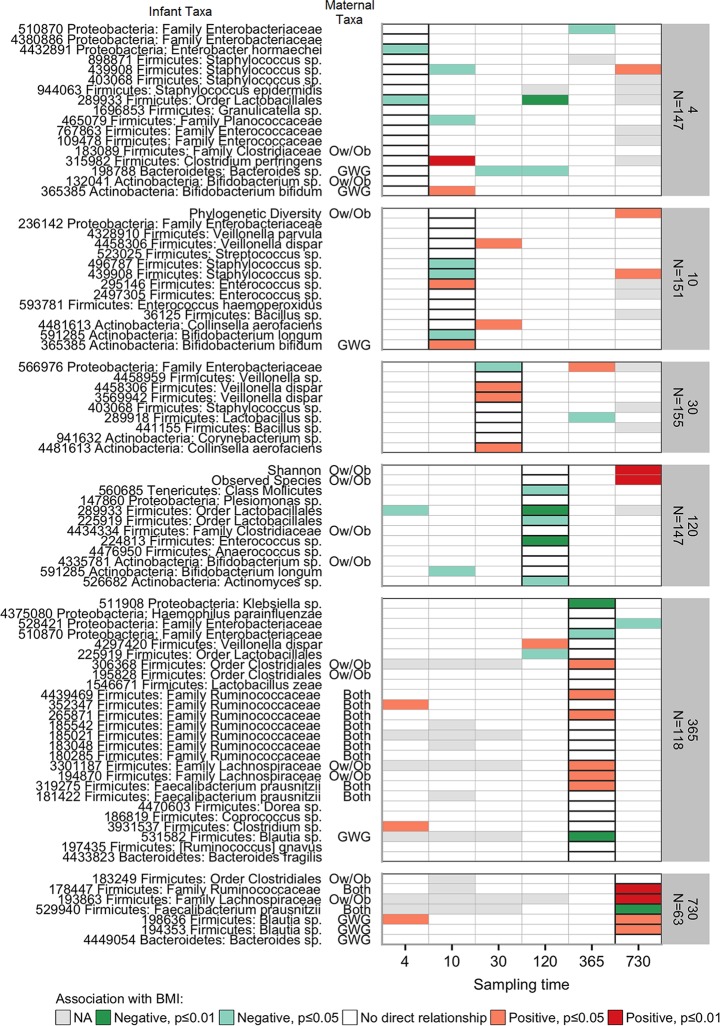
The infant gut microbiota taxa and alpha diversity measures at each sampling time that were most highly predictive of BMI z-score at age 12. Infant gut microbiota taxa and diversity measures (OTUs listed with OTU, phylum, and most specific level of known taxonomic classification) over the first 2 years of life that were selected as most predictive of childhood BMI z-score in random forests at six points (grouped vertically). We used linear regressions in order to assess the direction of association between these features at each sampling time with BMI z-score at age 12, controlling for confounding factors (breastfeeding, delivery mode, antibiotic exposures, gestational age at birth, and twin status). While a feature may have been selected as predictive of BMI only at a certain sampling time, we plotted the linear associations at all times (horizontal axis) in order to assess the temporal consistency of the association. Since the assumptions underlying random forests and linear regressions are very different, we would not expect important features to necessarily be significant in regressions. The colors represent the regression coefficients for each feature: red indicates a positive relationship between the feature and childhood BMI, e.g., higher abundance corresponds with higher BMI; green indicates negative relationships; gray indicates that the regression model failed to converge (NA). The column labeled “maternal taxa” shows whether these gut microbiota species or diversity measures were also associated with maternal overweight/obesity (Ow/Ob), excessive gestational weight gain (GWG), or both, in the maternal gut microbiota at the time of delivery; Fig. 5 provides more detail and Fig. S4 shows the association between these BMI-associated infant taxa and maternal characteristics.

The gut microbiota taxa and alpha diversity measures that were identified as most strongly predictive of later childhood BMI are shown in Fig. 3. The predictors in random forests can have complex interrelationships with each other and with the outcome, which makes them appropriate for gut microbiota data but challenging to interpret (23). Thus, we used adjusted linear regressions to aid in interpretation and specifically to evaluate whether any of these predictors showed a linear relationship with BMI (see Fig. S3 for conceptual model; see Table S1 for full regression results). Because the assumptions underlying random forests and linear regressions are very different, we would not expect important features to necessarily be significant in regressions; however, many of these predictors did show linear relationships with BMI.

10.1128/mBio.01751-18.5 TABLE S1 Results of unadjusted and adjusted linear regressions to assess the direction of association between infant gut microbiota features (selected as highly predictive of childhood BMI z-score) and BMI z-score. Adjusted models included the following: exclusive breastfeeding, delivery mode, antibiotics, twin status, and gestational age at birth. Download Table S1, XLSX file, 0.1 MB. Copyright © 2018 Stanislawski et al. 2018 Stanislawski et al. This content is distributed under the terms of the Creative Commons Attribution 4.0 International license.

10.1128/mBio.01751-18.4 FIG S4 The association between maternal (a) prepregnancy Ow/Ob and (b) excessive GWG and the infant gut microbiota taxa that were highly predictive of childhood BMI. This plot shows the strength of association between maternal characteristics and the infant gut microbiota taxa selected as highly predictive of childhood BMI at each sampling time (i.e., those shown in Fig. 3). The *P* values are from unadjusted (circles) and adjusted (squares) permutational ANOVA models of the unweighted (coral) and weighted (blue) UniFrac distance matrices of the taxa. Dotted lines show *P* = 0.05 (black) and *P* = 0.1 (gray). Adjusted models controlled for breastfeeding, delivery mode, antibiotic exposures, and gestational age at birth. Download FIG S4, TIF file, 0.4 MB. Copyright © 2018 Stanislawski et al. 2018 Stanislawski et al. This content is distributed under the terms of the Creative Commons Attribution 4.0 International license.

### Early-life BMI z-scores do not predict later childhood overweight/obesity.

**FIG 4:**
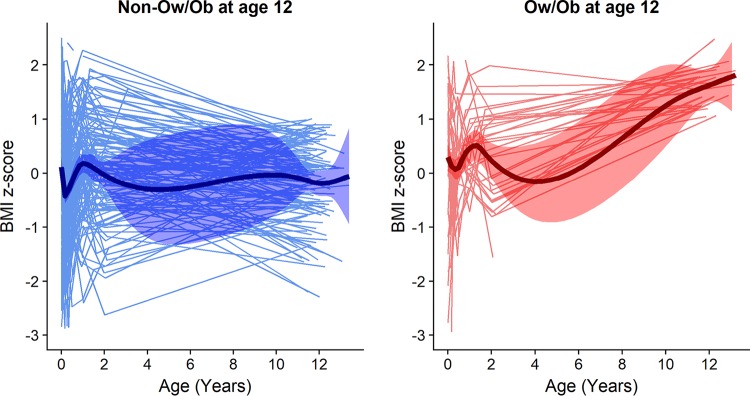
Spaghetti plots of BMI z-scores over time by Ow/Ob status at age 12. The regression lines (denoted by thicker lines) show that BMI z-scores were fairly constant between ages 2 and 12 for children who were not Ow/Ob at age 12, but there was an increase in BMI z-scores during this time for children who became Ow/Ob. At age 2, there was no significant difference between BMI z-scores of children who later became Ow/Ob and those who did not.

BMI z-scores showed considerable variation during the first 2 years of life (Fig. 4). Non-Ow/Ob children showed no significant change in BMI z-scores between early childhood and age 12 (β = 0.01; 95% CI: −0.01, 0.02; *P* value = 0.42), while Ow/Ob children showed an increase in BMI z-score (β = 0.13; 95% CI: 0.1, 0.15; *P* value < 0.001). BMI z-scores at age 2 years did not differ significantly by Ow/Ob status at age 12 (*P* value = 0.32). Among children who were classified as lean at age 12, 11.3% had a BMI z-score of ≥85th percentile (the cutoff used to define Ow/Ob) (24) at the age of 2, whereas only 4.2% met this cutoff among those who would become Ow/Ob. Thus, the overweight phenotype was largely absent at 2 years for the children who became Ow/Ob by age 12.

### Maternal gut microbiota taxa associated with maternal Ow/Ob and excessive GWG show substantial overlap at the species level with BMI-associated taxa in the infant.

**FIG 5:**
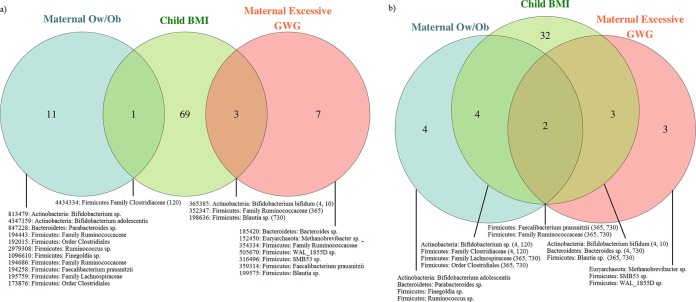
Venn diagrams showing the overlap between gut microbiota (a) OTUs and (b) species associated with maternal overweight/obesity (Ow/Ob) and excessive gestational weight gain (GWG) in the maternal taxa at the time of delivery and those predictive of childhood BMI in the infant gut microbiota taxa during the first 2 years of life. The OTUs are listed with OTU, phylum, and most specific level of known taxonomic classification; “species” are listed as phylum and most specific level of known taxonomic classification. The numbers in parentheses show at which time points in the infant these taxa were predictive of childhood BMI. Due to the large number of taxa associated with child BMI (green circle), these are not all listed by name but are shown in Fig. 3.

In prior work, we examined the association of maternal Ow/Ob and excessive GWG with maternal gut microbiota at the time of delivery in this cohort (25). We found that maternal Ow/Ob was associated with alpha diversity and taxonomic differences in composition, while excessive GWG was associated only with taxonomic differences. If the associations between maternal Ow/Ob or excessive GWG and child obesity are mediated by the gut microbiota, we might expect to see some overlap in the maternal taxa associated with these conditions and the infant taxa associated with childhood BMI. Figure 5 shows the overlap in the taxa associated with these maternal characteristics and those predictive of childhood BMI, at both the operational taxonomic unit (OTU) (Fig. 5a) and species (Fig. 5b) level; 1/12 (8%) of the maternal OTUs most associated with maternal Ow/Ob was also predictive of childhood BMI in the infant gut microbiota, while 3/10 (30%) of those associated with excessive GWG were predictive of BMI in the infant gut. When these taxa were summarized at the species level, there was even more overlap (Fig. 5b); 6/10 (60%) of the species most associated with maternal Ow/Ob and 5/8 (63%) of those associated with excessive GWG were predictive of BMI in the infant gut. Additionally, we highlighted which of the infant taxa associated with child BMI in Fig. 3 also associated with these maternal characteristics in maternal taxa at the time of delivery.

### BMI-associated infant gut microbiota and the association with maternal characteristics of prepregnancy Ow/Ob and excessive GWG.

We assessed the association between both maternal prepregnancy Ow/Ob and excessive GWG with the groups of infant gut microbiota taxa selected as most predictive of childhood BMI (i.e., those shown in Fig. 3) using permutational ANOVA (Fig. S4). Maternal Ow/Ob was associated with the qualitative differences in the selected infant gut microbiota taxa at day 30 (*P* value = 0.005 for unweighted UniFrac); excessive GWG was associated with quantitative differences at day 730 (*P* value = 0.019 for weighted UniFrac).

## DISCUSSION

In this study, we identified associations between infant gut microbiota composition at six time points during the first 2 years of life and the development of later childhood obesity. We found that the gut microbiota during the first 2 years of life, particularly at 2 years of age, was strongly associated with later childhood BMI. Interestingly, BMI z-scores at age two were not significantly higher in children who later became Ow/Ob, so development of the gut microbiota composition that predicted later BMI preceded any measurable excess weight in the children. In prior work in this cohort, we also observed that early gut microbiota was associated with infant growth rates (10) and that maternal Ow/Ob and excessive GWG were associated with compositional differences in maternal gut microbiota at the time of delivery, although these maternal characteristics were not associated with an overall characteristic taxonomic signature in the infant gut microbiota during the first 2 years of life (25). Here we show that the subset of the infant gut microbiota taxa that are associated with later childhood BMI were also associated with maternal Ow/Ob and excessive GWG. Furthermore, the taxa in the maternal gut microbiota that were most associated with these characteristics showed substantial overlap with the infant taxa most related to childhood BMI.

Our results showed that the gut microbiota at age two, when most of the children who later became Ow/Ob did not yet have high BMI z-scores, was highly predictive of BMI at age 12. One avenue for the prevention of obesity would be through early identification of individuals at high risk for development of obesity, and our findings suggest that fecal microbiota during early childhood may have potential as part of an obesity risk prediction algorithm, which could be particularly advantageous given the ease of recovering samples from diapers. Dietary or other interventions could be targeted to these individuals before they begin to gain weight. Our results are from a fairly homogeneous Norwegian cohort; thus, additional work would be necessary to extend our findings to other populations and to explore how the patterns vary with early-life exposures.

It is possible that the gut microbiota characteristics that we found to associate with later BMI reflect aspects of the environment, particularly dietary and/or lifestyle patterns, which may have independently influenced risk for obesity. Diet is one of several factors that influence the gut microbiota (26, 27), and the gut microbiota may mediate diet-induced obesity (5). The increasing association between gut microbiota at 1 and 2 years and later childhood BMI could thus be partly due to dietary factors that are precursors to obesity and that increasingly shape the infant gut microbiota at those ages. Taxa in the genus *Bacteroides* were selected as predictive of BMI in the 1- and 2-year gut microbiota, and this genus has been shown to be largely shaped by environmental factors, including diet (27, 28). Studies of diet-induced obesity in mice suggest that probiotics can protect against weight gain and metabolic abnormalities (29, 30). Thus, even in the context of a poor diet, interventions may still protect against weight gain.

Both maternal Ow/Ob and excessive GWG are predictors of obesity in children, both in this cohort and in many others (31, 32). The maternal gut microbiota may contribute toward offspring risk for obesity through vertical transfer, as well as through *in utero* effects (33, 34). There is strong evidence that many early infant gut taxa are transferred from the mother, particularly for certain taxa, including *Bifidobacterium* (35, 36). Strain-level similarity between mothers and infants decreases over time, but species-level composition converges (35). Our analysis of the overlap between maternal gut microbiota associated with Ow/Ob or excessive GWG and infant gut microbiota associated with childhood BMI reflects both early OTUs that may have been transferred from the mother (e.g., Bifidobacterium bifidum) and later-colonizing species that may reflect shared environmental exposures (e.g., *Blautia* sp.).

The putative environmental source of some of these BMI-associated infant gut microbiota taxa does not necessarily give us insight into whether they are predominantly a reflection of environmental factors that cause obesity or whether they are also driving metabolic programming. While transfer of strains into gnotobiotic mice could be an important future direction for resolving these causal links (as has been valuable in linking population-level data to causal mechanisms in adult BMI [37]), there is evidence to support the idea that environmentally derived microbes that colonize during a key time in development could have consequential long-term programming effects (14, 38). Furthermore, although the association between infant gut microbiota and childhood BMI became stronger as the infant aged, we did see associations within the first months of life as well, and it is unlikely that this is driven by lifestyle factors because the majority of babies in this cohort were exclusively breastfed, limiting the influence of diet, and physical activity would not yet be a factor at this age. There is also evidence that many of the taxa associated with BMI in our findings have immune or metabolic modulating effects, such as early proteobacteria, Faecalibacterium prausnitzii, and Bacteroides fragilis (39–41). We have limited understanding of the temporal interplay between gut microbiota taxa and the developing immune and metabolic systems in early life, which may be of key importance. While inflammation and proinflammatory gut microbiota are often associated with disease in adults (42), proinflammatory gut microbiota may be an essential aspect of immune system training in early life (39, 43). It is interesting to speculate about some of our findings in this context. For example, species of *Bifidobacterium* are generally thought to promote health, particularly in infants. Consistent with many prior studies, we found that early abundance of OTUs of *Bifidobacterium* sp. and Bifidobacterium longum was associated with lower BMI (7, 11, 44). However, we found that Bifidobacterium bifidum at 10 days showed the opposite association with BMI. While the positive association of B. bifidum at 10 days with childhood BMI seen in our results could also be spurious, these findings are concerning because strains of this species are included in many prenatal and infant probiotics. One of the few prior studies that likewise examined gut microbiota at numerous early time points in life found that infants with later acquisition of high levels of *Bifidobacterium* and *Collinsella* had lower adiposity at 18 months of age (9). Thus, it is possible that the timing of colonization by *Bifidobacterium* has important consequences for later adiposity, or that there are important strain-level differences in the effects of *Bifidobacterium* in early life. It is also possible that early colonization with higher levels of Bifidobacterium bifidum reflects an earlier transition from an aerobic to an anaerobic environment with a shorter colonization of important proinflammatory gut microbiota (9, 39).

Some of the associations between gut microbiota and childhood BMI seen in this study support previous obesity-related research findings. For example, F. prausnitzii abundance at 2 years predicted lower childhood BMI, as seen in adults (45). Higher *Streptococcus* in the first months of life has been associated with higher adiposity (9) and BMI (7), which is consistent with our results. Some prior studies have suggested that higher *Lactobacillus* and lower *Bacteroides* levels in the first 3 months of life are associated with higher risk for infant and child overweight (46), but we found the opposite associations with childhood BMI.

This study has some important limitations. The samples were weighted toward earlier time points, with fewer infants having samples at 1 and 2 years due to loss to follow-up. We did not have strain-level metagenomic data, so our analysis of similarity between mother-infant pairs is not definitive of vertical transfer. This cohort is almost entirely ethnically Norwegian, and the non-Norwegians were typically from other Nordic countries. Therefore, some of the taxonomic findings may not generalize to other ethnic or racial groups or geographic regions. However, the results should be internally consistent in terms of showing support for the notion that infant gut microbiota is associated with later BMI and that maternal Ow/Ob may play a role in shaping this composition. Obesity is a complex condition with complex etiology, and there have been notable inconsistencies across human studies in terms of the associations with gut microbiota (45, 47, 48). Some of these inconsistencies could be due to geographical, racial/ethnic, and genetic variation across study populations (49, 50). Thus, the homogeneity in our study may have allowed us to isolate an association between infant gut microbiota and later BMI that could be attenuated by population heterogeneity in other cohorts, which would need to be further studied and understood, as well as how these patterns vary across diverse early-life exposures. A primary strength of this study is the large cohort of infants with repeated gut microbiota samples during the first 2 years of life, maternal gut microbiota samples at the time of delivery, and the extensive data on maternal and infant characteristics and exposures, which were controlled for in the analyses.

Overall, our findings show a strong association between infant gut microbiota at age two and BMI at age 12 and show that the gut microbiota characteristics predictive of later BMI precede excessive weight gain, suggesting that the gut microbiota could have potential to help identify children at risk for obesity. We also found some support for the hypothesis that maternal Ow/Ob may influence some of the infant gut microbiota taxa that are associated with later BMI. Further studies of the specific bacteria highlighted in our results may also lead to greater understanding of the etiology of obesity.

## MATERIALS AND METHODS

### Study cohort.

NoMIC is a Norwegian birth cohort of 552 children designed to study the establishment of gut microbiota during infancy and its consequences for child health. Participating mothers, recruited between 2002 and 2005, were asked to fill out periodic questionnaires and to collect and freeze fecal samples from themselves at 4 days postpartum and from their infants at days 4, 10, 30, 120, 365, and 730 postbirth. Study personnel retrieved the fecal samples and kept them frozen during transport to the Biobank of the Norwegian Institute of Public Health, Oslo, where they were stored at −20°C upon arrival.

The study was approved by the Regional Ethics Committee for Medical Research in Norway (approval reference 2002, S-02216) and the Norwegian Data Inspectorate (reference 2002/1934-2). The approvals, as well as signed informed consent from the mothers, were obtained prior to collection of data and samples. The NoMIC study was funded by the FRIMEDBIO program at the Norwegian Research Council.

### Study sample.

This study includes 781 infant gut microbiota samples from 165 infants in NoMIC for whom later childhood height and weight were available (median age, 11.7 years; IQR, 11.4 to 12.3), as well as maternal height and prepregnancy weight. Samples were collected at days 4, 10, 30, 120, 365, and 730, with fewer samples at the 1- and 2-year sampling times (*n* = 147, 151, 155, 147, 118, and 63, respectively). In our current analyses, we compared some of our results to prior work in which we examined the association between maternal Ow/Ob (*n* = 169) and excessive GWG (*n* = 117) and maternal gut microbiota 4 days postpartum in NoMIC (25). A subset of these women (*n* = 71) are mothers of the 165 children included in our primary analysis, and their gut microbiota samples are included in the taxonomic plots (see Fig. S1 and S2 in the supplemental material).

### Data sources.

Height and weight were measured by a study nurse at follow-up examinations when the children were approximately 12 years of age (median = 11.7; IQR, 11.4 to 12.3; range, 10.8 to 13.4). Age- and sex-specific BMI z-scores and percentiles were calculated based on CDC growth charts (51). Childhood overweight and obesity were defined using these BMI percentiles according to CDC standards (overweight, ≥85th percentile; obese, ≥95th percentile) (24). This age was chosen for the outcome measure because it correlates with adult obesity more strongly than earlier measures of BMI (18).

Mothers also extracted information on infant height and weight from their “baby health visit” cards and reported this information in questionnaires, providing this information for numerous ages during childhood (range, 0 to 5 years; 99% of the measurements at ≤2 years; median number of measurements per child, 7; IQR, 6 to 8). As recommended by the CDC, age- and sex-specific BMI z-scores were calculated using WHO growth charts for ages under 2 years and CDC growth charts for ages over 2 years (51, 52).

Prepregnancy BMI was based on maternal self-report of weight at the first clinic visit of pregnancy; the median time of the first visit was at 9 weeks of gestation (IQR, 7.3 to 11.3 weeks). Height and weight were also measured at that visit. Prepregnancy BMI was initially categorized as: underweight, normal weight, overweight, and obese according to standard definitions (53). We then further combined these groups into (i) non-Ow/Ob (55.8%)—underweight (8.5%) and normal weight (47.3%)—and (ii) Ow/Ob (44.2%)—overweight (30.9%) and obese (13.3%).

The definition of maternal excessive GWG was previously described for the NoMIC cohort (25). Briefly, mothers who were missing GWG data or not full term were excluded because there are not well-established weight gain recommendations for preterm births. The recommended range of the Institute of Medicine (IOM) was used to define adequate GWG, which is based on prepregnancy BMI (54); weight gain more than this amount for the respective BMI group was considered “excessive.” GWG was calculated using the prepregnancy weight and final weight from self-report in a questionnaire approximately 1 month postdelivery.

Maternal questionnaires at 1, 6, 12, and 24 months provided information on mode of delivery, maternal age, education, parity, maternal smoking, ethnicity, infant sex, maternal and infant use of antibiotics, breastfeeding practices, and introduction of solid foods. We obtained information on gestational age at birth from the Medical Birth Registry of Norway.

### Processing of microbial samples.

DNA was extracted using standard protocols, as previously described for this cohort (55, 56). The extracted DNA was amplified using PCR with barcoded primers targeting the V4 region of the 16S rRNA gene. Sequences were generated using an Illumina HiSeq instrument (Illumina, San Diego, CA). A total of 96,632,013 good-quality sequencing reads were obtained; the median reads per sample were 111,943 (IQR, 80,207 to 155,887). Operational taxonomic units (OTUs) were assigned using UCLUST (57) as implemented in QIIME v1.9.1 (58) via a closed reference-based system using the Greengenes 13.8 (59) database and a 97% threshold. Across the maternal and infant gut microbiota samples, there were 7,832 OTUs. A rarefied OTU table at 5,000 sequences per sample served as input for the analyses.

### Statistical analysis.

We compared child demographic and birth characteristics by overweight/obese status at age 12 using chi-square tests for categorical variables and Wilcoxon rank sum tests for continuous variables. We used principal coordinate analysis plots of weighted and unweighted UniFrac (60) distance of the infant gut microbiota samples by sampling time, as well as the maternal gut microbiota samples near the time of delivery, to visualize the changes in the microbial communities in the samples with age. We evaluated the relationships between covariates and the infant gut microbiota using permutational ANOVA of the UniFrac (unweighted and weighted) distance matrices at each time point.

### Regressions and random forests to assess the association between infant gut microbiota and later childhood BMI z-score.

In order to examine the association between overall infant gut microbiota composition during the first 2 years of life and BMI z-scores at age 12, we first used Microbiome Regression-Based Kernel Association Tests (MiRKAT) (61) of the unweighted and weighted UniFrac distance matrices, which capture qualitative and quantitative differences in phylogeny, respectively. We then evaluated whether specific taxa at each sampling time associated with later BMI using VSURF (Variable Selection using Random Forests) (22) for feature selection at each sampling time. The VSURF function is a multistep algorithm that selects the most important features for the prediction of the outcome. We included all OTUs meeting the minimum threshold of presence in 10% of samples at that time and a minimum of 0.1% for the maximum relative abundance of each sample, in addition to four measures of alpha diversity: phylogenetic diversity (PD), Shannon diversity index, Chao1, and observed species. We also included the following potential confounding variables: exclusive breastfeeding (yes/no for samples ≤120 days; duration of exclusive breastfeeding for samples at later times), delivery mode, antibiotic exposure (yes/no at each time), twin status, and gestational age at birth. See the directed acyclic graphs (DAGs) in Fig. S3 in the supplemental material for a full description of the choice of adjustment variables for this and following analyses and the relationships between exposures and outcomes. We did not adjust for childhood diet, but this could not have affected the gut microbiota at the earliest stages when the child was still mainly breastfed (first 4 months). We also did not adjust for physical activity because until 2 years of age it would be unorganized and child controlled; anything after 2 years of age would not have affected the collected gut microbiota samples and thus would not meet the definition of a confounding factor.

We then used repeated cross-validation (3-fold, 100 repetitions) of random forests in the caret (62) package in order to evaluate the *R*^2^ of the selected features at each time to predict BMI z-score. This method involves repeatedly using a subset of samples as a training set and the remaining samples as the test set to predict the outcome. Since the confounding factors were not among the most important selected features for prediction of BMI, we estimated *R*^2^ both with and without these factors.

### Regressions to investigate the nature of the relationships between random forest selected gut microbes and later childhood BMI z-score.

Random forests are an ensemble technique that allow for complex interactions between the predictors. While all of the microbiota features (taxa and alpha diversity) selected by the VSURF tool may be important together to predict BMI, we also wanted to understand whether these features individually had linear associations with BMI at age 12. Thus, we used linear regression models for each sampling time with BMI z-score as the outcome and each feature selected by the random forests (described above) as the predictor. Since microbiota features are highly correlated, this method allowed us to assess the linear relationship with BMI for each feature. In these models, we controlled for the same potential confounding variables as used in the random forests: exclusive breastfeeding, delivery mode, antibiotics, twin status, and gestational age at birth. Since linear regressions were used as a secondary step to aid in the interpretation of the random forest results rather than as a tool for discovery applied to all taxa, multiple testing corrections were not applied to these results. The strength of association was indicated by direction of association and strength of evidence for a linear relationship: *P* ≤ 0.01, 0.01 ≤ *P* < 0.05, and no direct relationship.

### Longitudinal trends in BMI z-scores over infancy and childhood.

In order to assess whether BMI z-scores at age 12 were similar to those during infancy and early childhood, we plotted BMI z-scores by age using spaghetti plots. We additionally modeled the relationship using a mixed linear regression model with BMI z-scores as the outcome and a random intercept by child. The predictors included age, Ow/Ob status at age 12, and an interaction between age and Ow/Ob status. We used a *t* test to examine whether there was a significant difference in BMI z-scores at age 2 (range, 1.5 to 2.5 years) between children who were Ow/Ob at age 12 and those who were not for the subset of *n* = 104 children with BMI data during that time frame. For ages 2 to 18, overweight is defined as a BMI z-score percentile of ≥85% (24).

### Comparison of maternal gut microbiota taxa associated with maternal Ow/Ob and excessive GWG and the infant gut microbiota taxa associated with childhood BMI.

In prior work, we examined the association between maternal characteristics of prepregnancy Ow/Ob and excessive GWG with maternal gut microbiota taxa at the time of delivery (25). We used Venn diagrams to illustrate the overlap between the maternal taxa associated with these characteristics and the infant gut microbiota characteristics associated with childhood BMI. We examined these taxa at both the OTU level and the most specific assigned level of taxonomy.

### Association between maternal Ow/Ob and excessive GWG and BMI-associated infant gut microbiota.

In order to assess whether the gut microbiota characteristics identified as predictive of BMI at age 12 were also associated with exposure to maternal Ow/Ob or excessive GWG, we used permutational ANOVA of the UniFrac (unweighted and weighted) distance matrices for the selected infant gut microbiota features at each time point. Adjusted models controlled for exclusive breastfeeding, antibiotics, delivery mode at birth, and gestational age (see Fig. S3 for DAGs).

We used SAS v9.4 (SAS Institute Inc., Cary, NC), R v3.3.2 (63), and QIIME v1.9.1 (58) for analyses. *P* values less than 0.05 were considered statistically significant.

### Availability of data and materials.

Pursuant to the Norwegian Health Research Act and the Norwegian Data Protection Act, approval from the Regional Ethical Committees is required for use (and storage) of personal data related to health. The full data used for this study can thus be shared when a study protocol has been approved by the Norwegian Regional Ethical Committees and a data transfer agreement has been signed. Requests should be directed to Cathrine Thomsen (cathrine.thomsen@fhi.no) at the Norwegian Institute of Public Health. The raw sequences and limited personal information about participants, including age and sex, are available from the European Bioinformatics Institute (EBI), accession no. ERP111347.
