# Supplementary figures and images for: Gut Microbiota in the First 2 Years of Life and the Association with Body Mass Index at Age 12 in a Norwegian Birth Cohort
Source: mBio. 2018 Oct 23;9(5):e01751-18. doi: 10.1128/mBio.01751-18 (PMC6199494; doi:10.1128/mBio.01751-18)

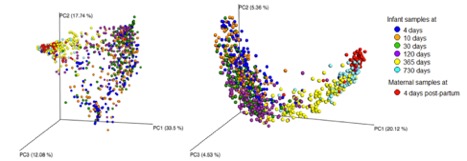

Supplement: FIG S2 [file mbo005184131sf2.jpg]

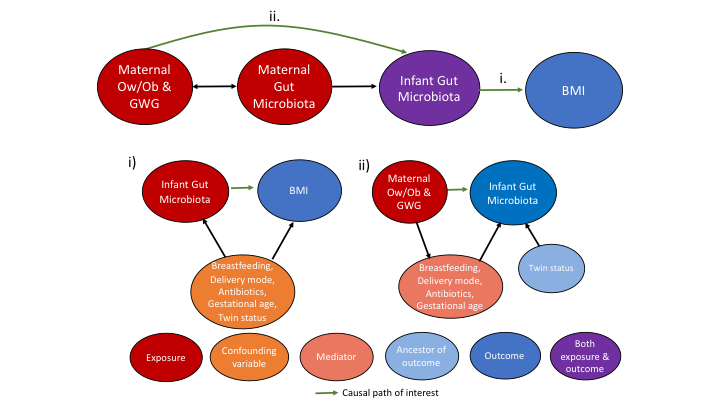

Supplement: FIG S3 [file mbo005184131sf3.tif]

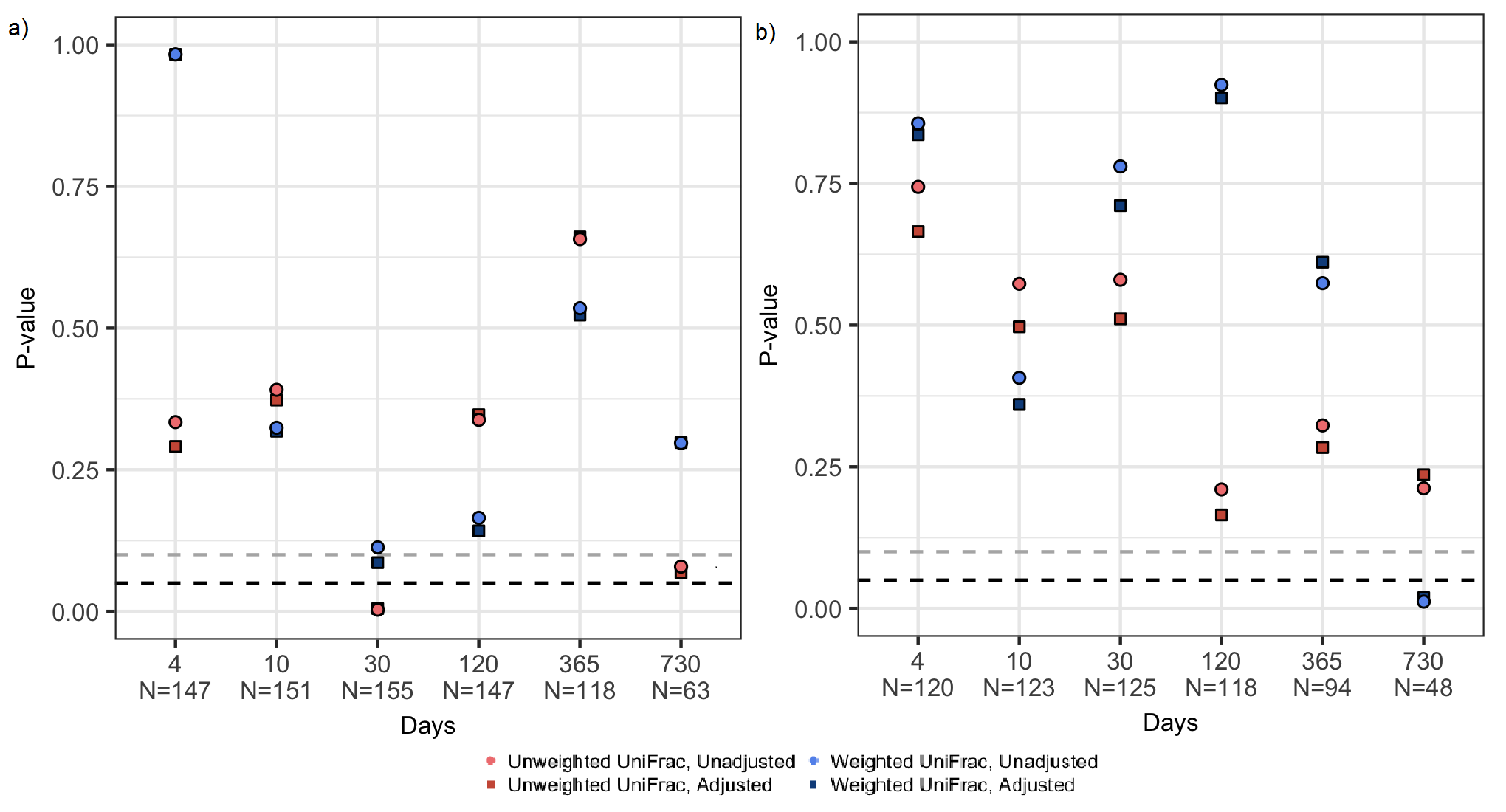

Supplement: FIG S4 [file mbo005184131sf4.tif]
